# A First Insight on the Population Structure of *Mycobacterium tuberculosis* Complex as Studied by Spoligotyping and MIRU-VNTRs in Santiago, Chile

**DOI:** 10.1371/journal.pone.0118007

**Published:** 2015-02-11

**Authors:** María Elvira Balcells, Patricia García, Paulina Meza, Carlos Peña, Marcela Cifuentes, David Couvin, Nalin Rastogi

**Affiliations:** 1 Infectious Diseases Department, School of Medicine, Pontificia Universidad Católica de Chile, Santiago, Chile; 2 Microbiology Laboratory, Clinical Laboratory Department, School of Medicine, Pontificia Universidad Católica de Chile, Santiago, Chile; 3 Respiratory Division and Microbiology Laboratory, Hospital San Borja Arriarán, Santiago, Chile; 4 WHO Supranational TB Reference Laboratory, Institut Pasteur de la Guadeloupe, Abymes, Guadeloupe, France; University of Padova, Medical School, ITALY

## Abstract

Tuberculosis (TB) remains a significant public health problem worldwide, but the ecology of the prevalent mycobacterial strains, and their transmission, can vary depending on country and region. Chile is a country with low incidence of TB, that has a geographically isolated location in relation to the rest of South American countries due to the Andes Mountains, but recent migration from neighboring countries has changed this situation. We aimed to assess the genotypic diversity of *Mycobacterium tuberculosis* complex (MTBC) strains in Santiago, Chile, and compare with reports from other Latin-American countries. We analyzed MTBC isolates from pulmonary tuberculosis cases collected between years 2008 and 2013 in Central Santiago, using two genotyping methods: spoligotyping and 12-loci mycobacterial interspersed repetitive unit-variable number of tandem repeats (MIRU-VNTRs). Data obtained were analyzed and compared to the SITVIT2 database. Mean age of the patients was 47.5 years and 61% were male; 11.6% were migrants. Of 103 strains (1 isolate/patient) included, there were 56 distinct spoligotype patterns. Of these, 16 strains (15.5%) corresponded to orphan strains in the SITVIT2 database, not previously reported. Latin American and Mediterranean (LAM) (34%) and T (33%) lineages were the most prevalent strains, followed by Haarlem lineage (16.5%). Beijing family was scarcely represented with only two cases (1.9%), one of them isolated from a Peruvian migrant. The most frequent clustered spoligotypes were SIT33/LAM3 (10.7%), SIT53/T1 (8.7%), SIT50/H3 (7.8%), and SIT37/T3 (6.8%). We conclude that LAM and T genotypes are the most prevalent genotypes of MTBC in Santiago, Chile, and together correspond to almost two thirds of analyzed strains, which is similar to strain distribution reported from other countries of Latin America. Nevertheless, the high proportion of SIT37/T3, which was rarely found in other Latin American countries, may underline a specific history or demographics of Chile related to probable human migrations and evolutions.

## Introduction

Tuberculosis (TB) is an infectious disease that remains present broadly despite intense global efforts for its control and elimination. In 2012, there were 8.6 million incident cases and more than 1.3 million deaths from tuberculosis worldwide [1]. Its causative agent, *Mycobacterium tuberculosis*, has accompanied human beings for thousands of years and, despite this very long period of coexistence, *M*. *tuberculosis* has remained among the most extreme examples of bacterial genetic homogeneity, with divergence rarely higher than 0.5 single nucleotide polymorphisms per genome per year [2][3]. Accordingly, for decades scientists have believed that different clinical or epidemiological outcomes of this infection in humans were largely related to host susceptibility and environmental factors, and not to the bacteria. However, this view has been challenged from the mid 2000’s, when comparative genomic and molecular epidemiological tools identified large sequence polymorphisms (LSPs) that allowed to differentiate *M*. *tuberculosis* into main six main lineages that defined their origin [4]. These lineages were defined as Indo-Oceanic (lineage I), East Asian (lineage II, including Beijing), East African Indian (lineage III), Euro-American (lineage IV), West-Africa (lineage V, M. *africanum* I) and West Africa (lineage VI; M. *africanum* II). Lineages I, V and VI were considered “ancient” and II to IV “modern” in relation to the presence or absence of the TbD1 genomic region, deleted in modern strains [4].

Independently in an earlier study, a distinction between three genetic groups of *M*. *tuberculosis* was achieved based on two non-synonymous polymorphisms occurring at high frequency in the genes encoding catalase-peroxidase and the A subunit of gyrase, which led to a classification in three principal genetic groups (PGG); group 1 bacteria being ancestral to groups 2 and 3 [5]. Additional genotyping methods such as the presence or absence of spacer oligonucleotides (spoligotyping), variable number of repeats (MIRU-VNTRs), or single nucleotide polymorphisms (SNPs), have not only provided further resolution at the sublineage level but also corroborated split of *M*. *tuberculosis* into similar main lineages; interested readers may refer to a recent review for comparison of spoligotyping-based nomenclature of *M*. *tuberculosis* lineages vs. PGG groupings, SNPs and SNP based cluster groups (SCGs), and LSP-based lineages [6].

*M*. *tuberculosis* molecular profiling allows today not only to study the global biodiversity and the phylogeographical variations of tubercle bacilli [6], but also to determine if recurrent disease is caused by endogenous reactivation or exogenous reinfection, to look for associations of particular *M*. *tuberculosis* strains with their clinical behavior, to determine whether outbreaks in specific geographical areas are caused by the same (cluster) or by multiple strains, if some strains are more transmissible than others, and whether some strains are more prone to develop drug resistance [7][8][9]. It undeniably constitutes an important tool for tuberculosis control and epidemiological studies today.

Chile has reached very low TB rates after decades of sustained decline related to economic development and a robust and well-coordinated National Tuberculosis Program. However, in the last 5 years TB incidence rate has become stationary around 13/100,000; with over 2000 new cases diagnosed per year. Newer policies will have to be addressed and implemented in the country to reverse this situation, such as strategies to detect earlier infections in high-risk groups, introducing contact tracing and targeted tuberculosis preventive therapy. In this context, the introduction of molecular epidemiology tools is a valuable asset to help understand local TB transmission dynamics. Two previous reports published locally have described genotyping of a small number of MTBC strains collected at the X^th^ region in year 2006 and Metropolitan Region in 2013, and the most frequently identified spoligotypes were T1 (24%) and LAM (39.5%), respectively [10, 11]. However, none of those reports included phylogenetic analysis or epidemiological data to distinguish local versus imported strains.

We hereby report on a preliminary study to describe genetic diversity and main genotypic lineages of *M*. *tuberculosis* complex clinical isolates circulating in Santiago, Chile, and compare our findings with MTB genetic epidemiology studies from other South American countries.

## Materials and Methods

### Patients and bacterial isolates

This study describes genotyping results of a total of 103 *M*. *tuberculosis* isolates collected from August 2008 to September 2013, the majority of strains (84.5%) between 2011 and 2013. The specimens were obtained from the same number of pulmonary TB patients, in 22 outpatient clinics and 3 large hospitals in the central area of Santiago city, with a reference TB incidence of 16.6 per 100,000 population for the year 2012. Our samples represent around 6.44% of all smear-positive TB cases for that period in the whole Metropolitan Region. Basic demographic data (age, gender and country of origin) was obtained from mandatory reports included in the National Tuberculosis Program registry and anonymized. Strains included newly and previously treated cases, and none of the samples were duplicated from a single patient. Culture processing and strain isolating were performed at the central clinical laboratories from the Hospital San Borja Arriarán and Red de Salud de la Pontificia Universidad Católica de Chile. Positive cultures grown over the Lowenstein—Jensen medium or in automatized liquid (MGIT) culture were isolated and DNA extracted by standard methods. Testing for susceptibility to first line drugs was determined over Lowenstein—Jensen medium using the proportion method [12] at the national reference laboratory (ISP). According to programmatic guidelines susceptibility testing was done in patients considered to be at risk for drug resistance, namely patients with TB treatment failure, default or relapse, exposed to a drug-resistant TB case, HIV-associated TB and cases in migrants from countries with high prevalence of drug resistant TB.

### Nucleic acid extraction and genotyping

*M*. *tuberculosis* complex species were confirmed for all strains by a specific PCR for gyrase B (gyrB) with primers described by Kasai et al. [13]. Briefly each amplification reaction contained: 12,5μl 2X Promega premix, 2μl of each primer 10μM (forward and reverse), 0,2 μl of GoTaq flexi 5U/μl (Promega) DNA polymerase, 5 μl of nuclease-free distilled water and 5 μl of DNA from samples or control, in a final volume of 25μl. Thermal cycling conditions were as follows: 95°C, 2 minutes followed by 10 cycles of 95°C, 30 seconds; annealing temperature step-downs every cycle of 1°C (from 65°C to 55°C); 72°C, 30 seconds. The annealing temperature for the final 35 cycles was 55°C with denaturation and extension phases as above. PCR products were analyzed through electrophoresis in 1.5% agarose gels that were stained with ethidium bromide.

Mycobacterial DNA was prepared by heat inactivation and lysis by sonication, and stored at-20°C until use. Spoligotyping analysis was performed by using a commercial source for membranes and reagents (Ocimum Biosolutions BV) as described by Kamerbeek et al. [14] [15]. A total of 43 spacers between the direct repeats in the target region were amplified by using DRa biotinylated at 5’ end and DRb primers. The PCR products were hybridized to a membrane containing 43 oligonucleotides by reverse line blotting. A positive control with *M*. *tuberculosis* H37Rv strain and *M*. *bovis* BCG were used in each run.

Standard 12-loci MIRU-VNTRs typing was manually performed by conventional PCR amplification of 12 of 41 loci: 02, 04, 10, 16, 20, 23, 24, 26, 27, 31, 39 and 40, as previously described [16]. Each locus was amplified separately by simplex PCR with GoTaq flexi DNA polymerase (Promega). Products were analyzed by electrophoresis using 1.5–2% agarose (Promega, Fitchburg, USA) gels. From the gel images, the corresponding MIRU-VNTR bands were interpreted as copy numbers based on a reference table [17].

### Computer assisted genotype analysis and comparison with databases

Spoligotype patterns as octal codes were entered in the SITVIT2 proprietary database of the Institut Pasteur de la Guadeloupe which is an updated version of the previously released SITVITWEB database (available online at http://www.pasteur-guadeloupe.fr:8081/SITVIT_ONLINE/) [18]. At the time of the analysis on August 2014, the database contained genotypes of about 112,000 *M*. *tuberculosis* clinical isolates from about 170 countries of origin. In this database, Spoligotype International Type (SIT) and MIRU International Type (MIT) designates patterns shared by two or more patient isolates, whereas “orphan” designates patterns reported for a single isolate [18] [19]. Genotypic lineages were assigned according to the rules described in SITVITWEB [18] in which the LSP-based Euro-American lineage (lineage IV) is split in Latin American and Mediterranean (LAM), ill-defined T, Haarlem (H), X, and S lineages. Importantly, the nomenclature “East-African Indian” (EAI or lineage III) denotes two altogether different *M*. *tuberculosis* clades by spoligotyping vs. LSPs [6]; EAI by spoligotyping was named “Indo-Oceanic” by LSPs, while EAI by LSPs corresponds to Central-Asian (CAS) in the SITVIT2 database [6]. Note that lineages could be subdivided into sublineages.

Data were also analyzed using MIRU-VNTRplus web server (http://www.miru-vntrplus.org/) which is a collection of 186 strains representing the major *M*. *tuberculosis* complex lineages [19][20]. This database contains information of strain species, lineage, country together with copy numbers of 24 MIRU loci, spoligotyping patterns, regions of difference (RD) profiles, single nucleotide polymorphisms (SNPs), drug susceptibility data, and IS*6110*-RFLP fingerprint images.

### Phylogenetic analysis

BioNumerics software version 6.6 (Applied Maths, Sint-Martens-Latem, Belgium) was used to compare spoligotypes and MIRU-VNTRs patterns, by drawing Minimum spanning trees (MSTs) in order to visualize evolutionary relationships between the clinical isolates in our study. MSTs are undirected graphs in which all samples are connected together with the fewest possible connections between nearest neighbors. An Unweighted Pair Group Method with Arithmetic mean (UPGMA) tree was also constructed using MLVA Compare software (http://www.ridom.de/mlvacompare/) to visualize deeper associations between spoligotypes and MIRU-VNTRs involved in this study.

### Ethics Statement

Prior to the start of the study, ethical approval was obtained from Ethical Committee of Pontificia Universidad Católica de Chile School of Medicine (CEC-MedUC). The microbiological records and basic demographic information of patients were anonymized and de-identified prior to analysis. Therefore, informed consent was not required and was specifically waived by the Ethical Committee.

## Results

In total, 103 *M*. *tuberculosis* isolates were collected and analyzed; patients providing samples were 61.2% males and 38.8% females, with a mean age of 45.3 (range 3–93) and 51.05 years old (range 22–93), respectively. Although all patients were current residents in Santiago, Chile, 11.6% were foreign-born migrants with main country of origin being Peru (66.6%) and Bolivia (25%).

Spoligotyping of the 103 isolates revealed a total of 56 different patterns: 16 patterns (15.5%) corresponded to orphan strains in the SITVIT2 database, not yet reported, and 40 patterns (containing 87 strains) corresponded to shared-types or SITs in the SITVIT2 database. Among these 40 shared-types, 2 isolates from the present study in Chile matched an orphan strain recorded in the database from USA, therefore a new shared-type, SIT4013, was created. In addition, a total of ten 12-loci MIRU patterns (from MIT 1670 to MIT 1679) were newly created. Description of the 40 SITs (n = 87 isolates) and corresponding spoligotyping defined lineages/sublineages is shown in Table 1. Among orphan isolates, we noted similar spoligotyping patterns (with absence of spacer 6) among orphans Or08, Or11, and Or12 within our study. (S1 Table)

**Table 1:** Description of 40 shared-types (SITs; n = 87 isolates) and corresponding spoligotyping defined lineages/sublineages starting from a total of 103 *M*. *tuberculosis* strains isolated in Santiago, Chile.

SIT*	Spoligotype Description	Octal Number	Nb. In study (%)	% in study vs. database	Lineage**	Unique vs. Clustered SIT***
1	□□□□□□□□□□□□□□□□□□□□□□□□□□□□□□□□□□■■■■■■■■■	000000000003771	2 (1.94)	0.02	Beijing	Clustered
20	■■□■■■■■■■■■■■■■■■■■□□□□■■■■■■■■□□□□■■■■■■■	677777607760771	1 (0.97)	0.11	LAM1	Unique
33	■■■■■■■■□□□■■■■■■■■■□□□□■■■■■■■■□□□□■■■■■■■	776177607760771	11 (10.68)	0.84	LAM3	Clustered
34	■■■■■■■■□□■■■■■■■■■■■■■■■■■■■■■■□□□□■■■■■■■	776377777760771	2 (1.94)	0.23	S	Clustered
37	■■■■■■■■■■■■□■■■■■■■■■■■■■■■■■■■□□□□■■■■■■■	777737777760771	7 (6.8)	1.34	T3	Clustered
39	■■■■■■■■■■■■■■■■■■□■■■□□■■■■■■■■□□□□■□□■■■■	777777347760471	2 (1.94)	1.35	T4-CEU1	Clustered
42	■■■■■■■■■■■■■■■■■■■■□□□□■■■■■■■■□□□□■■■■■■■	777777607760771	7 (6.8)	0.2	LAM9	Clustered
49	■■■■■■■■■■■■■■■■■■■■■■■■■■■■■■□■□□□□■■■□■■■	777777777720731	1 (0.97)	0.39	H3	Unique
50	■■■■■■■■■■■■■■■■■■■■■■■■■■■■■■□■□□□□■■■■■■■	777777777720771	8 (7.77)	0.2	H3	Clustered
52	■■■■■■■■■■■■■■■■■■■■■■■■■■■■■■■■□□□□■■■□■■■	777777777760731	3 (2.91)	0.31	T2	Clustered
53	■■■■■■■■■■■■■■■■■■■■■■■■■■■■■■■■□□□□■■■■■■■	777777777760771	9 (8.74)	0.14	T1	Clustered
58	■■■■■■■■■■■■■■■■■■■□■■□■■■■■■■■■□□□□■■■■■■■	777777557760771	1 (0.97)	0.52	T5-Madrid2	Unique
64	■■■■■■■■■■■■■■■■■■■■□□□□■■■■□■■■□□□□■■■■■■■	777777607560771	1 (0.97)	0.25	LAM6	Unique
91	■■■□□□□□□□□□□■■■■□■■■■■■■■■■■■■■□□□□■■■■■■■	700036777760771	1 (0.97)	0.28	X3	Unique
161	■■■■■■■■■■■■■■■■■■■■□□□□■■■■■■■□□□□□■■■■■■■	777777607740771	1 (0.97)	7.69	LAM9	Unique
180	■■□■■■■■■■■■■■■■■■■■■■■■■■■■■■□■□□□□■■■■■■■	677777777720771	1 (0.97)	1.67	H3	Unique
203	■■■□□□□■■■■■■■■■■■■■■■□□□□□□□□□□□□□■■■■■■■■	703777740001771	1 (0.97)	4.76	CAS	Unique
211	■■■■■■■■□□□■□■■■■■■■□□□□■■■■■■■■□□□□■■■■■■■	776137607760771	3 (2.91)	2.83	LAM3	Clustered
219	■■■■■■■■■■■■■□□□□□■■■■■■■■■■■■■■□□□□■■■■■■■	777740777760771	1 (0.97)	1.16	T1	Unique
222	■■■■■■■■■■■■■■■■□□□□□■■■■■■■□■■■□□□□■■■■■■■	777774077560771	2 (1.94)	2.06	Unknown	Clustered
281	■■■■■■■■■■■■■■■■□■■■■■■■■■■■■■■■□□□□■■■■■■■	777775777760771	1 (0.97)	3.23	T1	Unique
336	■■■■■■■■■■■■■■■■■□■■■■■■■■■■■■■■□□□□■■■□■■■	777776777760731	1 (0.97)	0.85	X1	Unique
373	■■■■■■■■■■■■■■■■■■■■■■■□■■■■■■■■□□□□■■■■■■■	777777767760771	1 (0.97)	1.47	T1	Unique
390	■■■■■■■■■■■■■■■■■■■■■■■■■■■■■□□■□□□□■■■■■■■	777777777620771	1 (0.97)	3.03	H3	Unique
452	■■■■■■■■■■■■■■■■■■■■□□□□■■□□■■■■□□□□■■■■■■■	777777606360771	1 (0.97)	5.88	LAM9	Unique
738	■■■■■■■■■■■■■■■■■■■■□□□□■■■■■■■■□□□□■■■■■□□	777777607760760	1 (0.97)	16.67	LAM9	Unique
746	■■■■■■■■■■■■■■■■■■■■■■■■■■■■□■□■□□□□■■■■■■■	777777777520771	1 (0.97)	2.86	H3	Unique
948	■■■■■■■■■■■■■■■■■■■■■■■□□□□□□□□■□□□□■■□□□■■	777777760020611	1 (0.97)	8.33	H3	Unique
1277	■■■■■■■■■■■■■■■■■■□■□□□□■■■■■■■■□□□□■■■■■■■	777777207760771	2 (1.94)	6.67	LAM9	Clustered
1293	■■□■■■■■□□□■■■■■■■■■□□□□■■■■■■■■□□□□■■■■■■■	676177607760771	1 (0.97)	12.5	LAM3	Unique
1355	■■■■■■■■■■■■■■■■■■■□□□□□■■■■□■■■□□□□■■■□■■■	777777407560731	1 (0.97)	0.54	LAM	Unique
1474	■■□■■■■■■■■■■■■■■■■■□□■□■■■■■■■■□□□□■■■■■■■	677777627760771	1 (0.97)	33.33	T1	Unique
1624	■■■■■■■■□□□■■■■■■■■■□□□□■■■■□■■■□□□□■■■■■■■	776177607560771	1 (0.97)	16.67	LAM	Unique
1877	■■■□■■■■■□■■■■■■■■■■■■■■■■■■■■■■□□□□■■■■■■■	737377777760771	1 (0.97)	7.14	T1	Unique
1914	□■■■■■■■■■■■■■■■■■■■□□□□■■■■□■■■□□□□■■■■■■■	377777607560771	1 (0.97)	16.67	LAM6	Unique
2030	■■■■■■■■■■■■■■■■■■■■■□□□■□□□□□□■□□□□■■■■□■■	777777704020751	1 (0.97)	33.33	H1	Unique
2273	■■■■■■■■■■■■■■■■■■■■□□□□■■■■■■□■□□□□■■■□■■■	777777607720731	1 (0.97)	25	H3	Unique
2623	■■■□□□□□□□□□□□□□□□□□□□□□■□□□□□□■□□□□■■■■■■■	700000004020771	1 (0.97)	33.33	H1	Unique
2745	■□■■■■■■■■■■■■■■■■■■□□□□■■■■■■■■□□□□■■■■□■■	577777607760751	1 (0.97)	33.33	LAM9	Unique
4013*	■■■■■■■■■■■■■■■■■■■■■■■■■■■■■□□□□□□□■□□■■■■	777777777600471	2 (1.94)	66.67	Unknown	Clustered

* A total of 39/40 SITs containing 85 isolates matched a preexisting shared-type in the database, whereas 1/40 SIT (n = 2 isolates) was newly created. A total of 13/40 SITs containing 60 isolates were clustered within this study (2 to 11 isolates per cluster) while 27/40 SITs containing 27 strains were unique (for total unique strains, one should add to this number the 16 orphan strains, which brings the number of unclustered isolates in this study to 43/103 or 41.75%, and clustered isolates to 60/103 or 58.25%). Note that SIT followed by an asterisk indicates “newly created” SIT due to 2 or more strains belonging to an identical new pattern within this study or after a match with an orphan in the database; SIT designation followed by number of strains: 4013* this study n = 2, USA n = 1.

** Lineage designations according to SITVIT2 using revised SpolDB4 rules; “Unknown” designates patterns with signatures that do not belong to any of the major lineages described in the database.

*** Clustered strains correspond to a similar spoligotype pattern shared by 2 or more strains “within this study”; as opposed to unique strains harboring a spoligotype pattern that does not match with another strain from this study. Unique strains matching a preexisting pattern in the SITVIT2 database are classified as SITs, whereas in case of no match, they are designated as “orphan”.

Distribution of main lineages for the 103 isolates according to SITVIT analysis, showed that 35 (34%) belonged to the LAM family; 34 (33%) belonged to the ill-defined T super-family; 17 (16.5%) belonged to the Haarlem (H) lineage; 3 (2.9%) belonged to the X lineage; 2 belonged to the S lineage and 2 (1.9%) belonged to the Beijing lineage. The rest belonged to “Unknown” or single strain lineages, and included genotypes such as Bovis, CAS, and AFRI (Table 2). Among all LAM strains (n = 35), the main sublineages were LAM3 (42.9%), LAM9 (40%) and LAM6 (8.6%). Among the ill-defined T super-family (n = 34) its prototype T1 (58.8%) was the 1^st^ most frequent pattern in our study, followed by T3 (20.6%). The H lineage represented 16.5% (n = 17) of isolates subdivided in H3 (88.2%), and H1 (11.8%) sublineages. Diversity of strains among migrants (n = 12) were much higher and included lineages such as Beijing and CAS, which were uncommon among non-migrants. Comparison of lineage allocation by SITVIT and MIRU-VNTRplus was highly concordant for the majority of strains with the exception of T lineages: 27 out of 34 T strains on SITVIT analysis were reclassified by MIRU-VNTR, mostly as H (40.7%) and LAM (22.2%) (data shown in S2 Table).

**Table 2:** Distribution of main lineages according to SITVIT2 starting from a total of 103 *M*. *tuberculosis* strains isolated in Santiago, Chile.

Lineage	Number of strains (%)
AFRI	1 (0.97)
Beijing	2 (1.94)
BOV	1 (0.97)
CAS	1 (0.97)
H	17 (16.50)
LAM	35 (33.98)
S	2 (1.94)
T	34 (33.01)
X	3 (2.91)
Unknown	7 (6.80)

Spoligotype analysis identified clustering (of 3 or more isolates) among 48 of 103 isolates, whereas with MIRU12 identified clustering (of 3 or more isolates) among only 14 of 103 strains. As shown in Table 3, the most frequently clustered spoligotypes were SIT33/LAM3 (n = 11, 10.7%), SIT53/T1 (n = 9, 8.8%), SIT50/H3 (n = 8, 7.8%) and SIT37/T3 (n = 7, 6.8%). The high presence of SIT37/T3 (6.8% of strains) was noteworthy as this SIT is usually more commonly found in Eastern Africa, and migration from Africa is highly rare in Chile. With respect to 12-loci MIRU clustering, the most frequently clustered isolate was 12-MIT585 (n = 6), that in SITVIT2 database corresponds to spoligotypes SIT37/T3 (n = 5) and SIT281/T1 (n = 1). Furthermore, in the SITVIT2 database, 12-MIT585 was present in 6 isolates only: in Spain from two South American patients (from Ecuador and Peru, respectively); and in USA (n = 1), Caribbean (n = 2), and Belgium (n = 1).

**Table 3:** Description of clusters containing 3 or more isolates in this study, and their worldwide distribution in the SITVIT2 database.

SIT (Lineage) Octal Number Spoligotype Description	Number (%) in study	% in study vs. database	Distribution in Regions with ≥3% of a given SIT*	Distribution in countries with ≥3% of a given SIT**
33 (LAM3) 776177607760771	11 (10.68)	0,84	AMER-S 34.96, AFRI-S 24.81, AMER-N 12.14, EURO-S 10.84, EURO-W 6.26, AMER-C 4.12	ZAF 24.81, PER 16.95, USA 12.14, BRA 10.92, ESP 6.79, ARG 4.35, FXX 4.05, ITA 3.51, HND 3.28
■■■■■■■■□□□■■■■■■■■■□□□□■■■■■■■■□□□□■■■■■■■				
53 (T1) 777777777760771	9 (8.74)	0,14	EURO-W 15.0, AMER-S 14.35, AMER-N 12.93, EURO-S 9.02, EURO-N 7.17, ASIA-W 7.0, AFRI-S 4.76, AFRI-E 4.46, ASIA-E 4.09, AFRI-N 3.37, EURO-E 3.13, CARI 3.1, AMER-C 3.1	USA 12.65, FXX 7.55, BRA 5.62, ITA 5.11, ZAF 4.65, PER 3.74, TUR 3.33, AUT 3.28
■■■■■■■■■■■■■■■■■■■■■■■■■■■■■■■■□□□□■■■■■■■				
50 (H3) 777777777720771	8 (7.77)	0,2	AMER-S 26.08, EURO-W 14.98, AMER-N 14.98, EURO-S 9.84, CARI 4.96, EURO-E 4.71, EURO-N 4.66, AFRI-N 3.63, AFRI-S 3.45, AFRI-M 3.22	USA 14.96, PER 13.65, BRA 7.15, FXX 5.87, AUT 5.19, ITA 4.63, ESP 4.63, ZAF 3.45, CMR 3.17, CZE 3.12
■■■■■■■■■■■■■■■■■■■■■■■■■■■■■■□■□□□□■■■■■■■				
37 (T3) 777737777760771	7 (6.8)	1,34	AFRI-E 19.19, EURO-N 11.9, EURO-W 11.52, ASIA-W 10.56, AMER-S 9.21, AMER-N 8.64, ASIA-E 6.14, EURO-S 5.76, EURO-E 4.42, ASIA-S 3.65, AFRI-S 3.26	ETH 16.7, USA 7.68, SWE 5.18, SAU 4.99, CHN 4.99, FXX 4.22, ITA 4.03, BRA 3.65, ZAF 3.26, DNK 3.26
■■■■■■■■■■■■□■■■■■■■■■■■■■■■■■■■□□□□■■■■■■■				
42 (LAM9) 777777607760771	7 (6.8)	0,2	AMER-S 31.16, AMER-N 11.33, EURO-S 10.81, EURO-W 9.04, AFRI-N 8.19, EURO-N 4.68, CARI 4.05, AMER-C 3.4, AFRI-E 3.4, AFRI-S 3.02	BRA 13.07, USA 11.33, COL 7.28, MAR 6.73, ITA 6.25, FXX 4.82, PER 3.54, ESP 3.2, VEN 3.17, ZAF 3.02
■■■■■■■■■■■■■■■■■■■■□□□□■■■■■■■■□□□□■■■■■■■				
52 (T2) 777777777760731	3 (2.91)	0,31	EURO-W 18.95, ASIA-E 14.97, EURO-N 13.4, AMER-N 11.62, EURO-S 5.13, ASIA-W 5.13, AFRI-M 5.13, AFRI-E 4.82, EURO-E 4.19, AMER-C 3.35, AMER-S 3.04	CHN 11.62, USA 11.2, SWE 9.42, FXX 8.69, BEL 4.71, CMR 4.19, ITA 3.35, JPN 3.14, ETH 3.14
■■■■■■■■■■■■■■■■■■■■■■■■■■■■■■■■□□□□■■■□■■■				
211 (LAM3) 776137607760771	3 (2.91)	2,83	AMER-N 30.19, AMER-C 20.76, EURO-W 19.81, AMER-S 18.87, EURO-S 7.55	USA 30.19, MEX 20.76, FXX 18.87, BRA 11.32, ESP 4.72
■■■■■■■■□□□■□■■■■■■■□□□□■■■■■■■■□□□□■■■■■■■				

* Worldwide distribution is reported for regions with more than 3% of a given SITs as compared to their total number in the SITVIT2 database. The definition of macro-geographical regions and sub-regions (http://unstats.un.org/unsd/methods/m49/m49regin.htm) is according to the United Nations; Regions: AFRI (Africa), AMER (Americas), ASIA (Asia), EURO (Europe), and OCE (Oceania), subdivided in: E (Eastern), M (Middle), C (Central), N (Northern), S (Southern), SE (South-Eastern), and W (Western). Furthermore, CARIB (Caribbean) belongs to Americas, while Oceania is subdivided in 4 sub-regions, AUST (Australasia), MEL (Melanesia), MIC (Micronesia), and POLY (Polynesia). Note that in our classification scheme, Russia has been attributed a new sub-region by itself (Northern Asia) instead of including it among rest of the Eastern Europe. It reflects its geographical localization as well as due to the similarity of specific TB genotypes circulating in Russia (a majority of Beijing genotypes) with those prevalent in Central, Eastern and South-Eastern Asia.

** The 3 letter country codes are according to http://en.wikipedia.org/wiki/ISO_3166-1_alpha-3; countrywide distribution is only shown for SITs with ≥3% of a given SITs as compared to their total number in the SITVIT2 database. Note that FXX code designates Metropolitan France.

**Fig 1:**
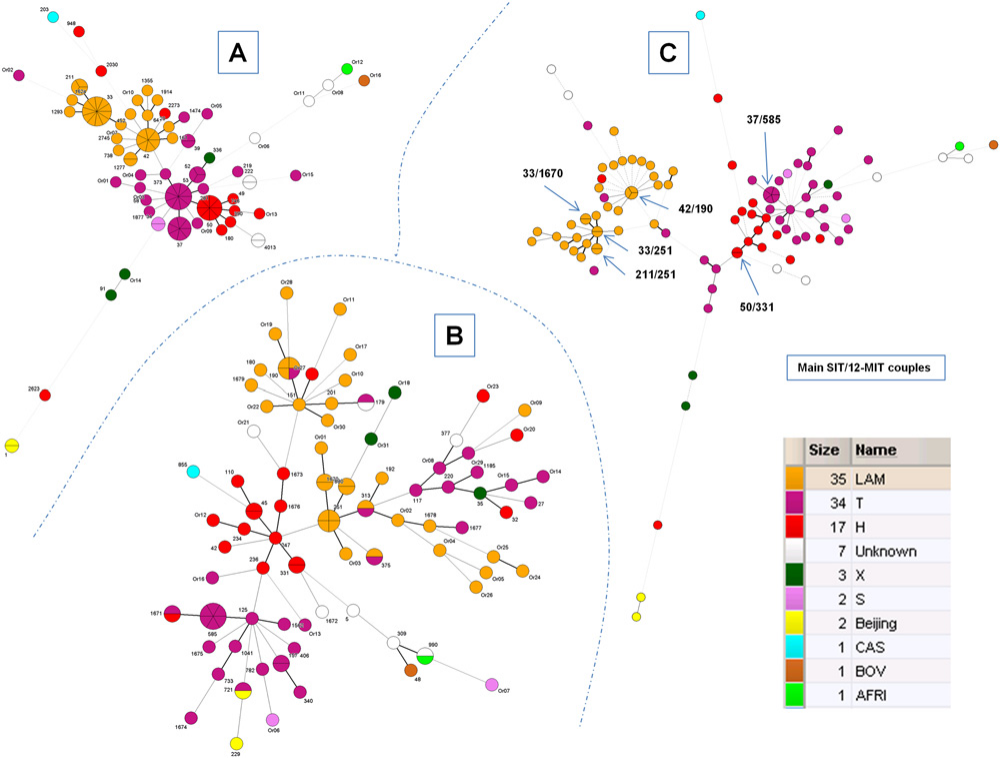
A minimum spanning tree (MST) illustrating evolutionary relationships between spoligotypes and 12-loci MIRU-VNTRs in Santiago, Chile (n = 103 isolates). **(A)** MST constructed on all spoligotypes; **(B)** MST constructed on all 12-loci MIRUs alone; and **(C)** MST constructed on the combination of spoligotypes and 12-loci MIRUs. The phylogenetic tree connects each genotype based on degree of changes required to go from one allele to another. The structure of the tree is represented by branches (continuous vs. dashed and dotted lines) and circles representing each individual pattern. Note that the length of the branches represents the distance between patterns while the complexity of the lines (continuous, gray dashed and gray dotted) denotes the number of allele/spacer changes between two patterns: solid lines, 1 or 2 or 3 changes (thicker ones indicate a single change, while the thinner ones indicate 2 or 3 changes); gray dashed lines represent 4 changes; and gray dotted lines represent 5 or more changes. The size of the circle is proportional to the total number of isolates in our study, illustrating unique isolates (smaller nodes) versus clustered isolates (bigger nodes). The separation inside circle also indicates the number of strains. The color of the circles indicates the phylogenetic lineage to which the specific pattern belongs. The labels of nodes indicate SITs and 12-MITs respectively in Fig. A and B; and main SIT/12-MIT couples (n ≥ 2 isolates) are represented in Fig. C.

Minimum spanning trees (MSTs) illustrating evolutionary relationships between spoligotypes and 12-MIRU-VNTRs were constructed for all strains included in the present study (n = 103). Fig. 1A shows a MST based on spoligotypes in which three major groups belonging to the modern group PGG2/3 (LAM, T, H, X, S) were evident as highly predominant (representing 88.3% of all strains), most notably LAM (n = 35, 34%), T (n = 34, 33%) and Haarlem (n = 17, 16.5%). More distance was evident among isolates belonging to the T sublineages than among those integrating the LAM sublineages (Fig. 1A). In contrast, when MST was constructed using 12-loci MIRU-VNTRs (Fig. 1B), isolates belonging to LAM appeared more distant than other sublineages, and for T genotype, two distinct and clearly separated groups were observed (Fig. 1B). The MST combining spoligotyping and MIRU-VNTRs results (Fig. 1C) highlights main SIT/12-MIT couples—in particular a SIT37/12-MIT585 cluster (n = 5 strains) and a SIT42/12-MIT190 cluster (n = 3 strains), as well as other smaller clusters containing 2 isolates. Briefly, there was an overall agreement in the manner in which the two genotyping methods grouped isolates in major lineages/sublineages. The UPGMA tree based on the combination of spoligotypes and 12-loci MIRUs using MLVA Compare (S1 Fig.) further corroborated the observations made.

Susceptibility data was available for 25/103 (24.3%) of all strains and revealed 1 case of multi-drug resistant (MDR) tuberculosis, 2 cases of rifampicin resistance and 2 cases of isoniazid monoresistance. No correlation with strain origins was possible due to the small number of resistant strains.

## Discussion

In the present study, we provided the first insight into the population structure of *M*. *tuberculosis* isolates in Santiago, Chile, showing the predominance of both the LAM and T lineages. This lineage distribution does not differ significantly as compared to descriptions reported from other Latin American countries where predominant isolates usually belong to three major clades: LAM, T, and Haarlem; in different proportions depending on specific country and year of isolation, but with generally a clear predominance of LAM (Table 4). Thus, in Brazil, the predominant *M*. *tuberculosis* lineage isolated between 1996 and 2005 was LAM (46%); followed by T (18.6%) and Haarlem (12.2%) families [21]. Similarly, in Argentina between years 2006 and 2007, the most common lineages were also LAM (50%), followed by T1 (18%) and Haarlem 1 (10%) and in Colombia, for strains isolated between years 1995 and 2007, LAM was present in 49.3% of isolates; Haarlem, in 25% and T group, in 13.8% [22] [23]. On the contrary, in Peru, which is Chile’s northern neighbor country, this proportion is lost as LAM strains have been found in lower percentage ranging from only 12.6 to 33.3% [24] [25] [26] [27].

**Table 4:** Main published studies (2006–2013) reporting distribution of *Mycobacterium tuberculosis* lineages in Central and South America.

Country, Period (years) and Reference	Numbe of strains	Harleem (H)	T	LAM	S	U	Beijing	X	EAI	Orphans or Unclassified
Argentina, 2006–2007 [22]	157	37%	18%	23%	NR	NR	NR	NR	NR	NA
		H1 10%	T1 18%	LAM9 13%						
		H2 16%		LAM3 10%						
		H3 11%								
Argentina, 2003–2009 [51]	787 (MDR only)	36.3%	13.9%	38.8%	2.8%	1.7%	1.5%	0.9%	NR	3.8%
			T1 8.9%	LAM3 16.4%						
Argentina, Brasil, Chile, Colombia, Venezuela, 2004–2008 [52]	951 (MDR only)	28.9%	17.4%	37.2%	NR	NR	1.3%	NR	NR	NR
French Guyane, 1994–2003 [34]	744	21.7%	30.1%	19.7%	1.5%	NR	1.0%	6.5%	4.8%	6.5%
Brasil, 1996–2005 [21]	1991	12.2%	18.6%	46%	1.9%	NR	NR	4.7%	0.85%	15%
				LAM9 10.3%						
Colombia 1995–2007[23]	152	25%	13.8%	49.3%	3.3%	NR	0.6%	1.3%	NR	6.6%
Colombia 2005–2008 [33]	414	44.3%		38.5%			0			
		H1 23.4%		LAM9:29.9%						
Paraguay, 2003 [39]	220	18.2%	8.6%	52.3%	9.5%	NR	0.5%	0.9%	NR	NR
Peru, 1996–2004 [24]	391 (MDR only)	11.4%	27.5%	33.3%	NR	0.3%	4.9%	4.4%	NR	18%
		H1 5.7%	T1 22.1%	LAM5 12.2%				X3 4.4%		
		H3 5.7%	T2 4.9%	LAM9 9.9%						
			T5 0.5%	LAM1 6.5%						
Peru, 2004–2006 [25]	323	23.8%	22.3%	23.8%	NR	NR	9.3%	NR	NR	13.3%
Peru, 2009 [26]	199	34.9%	15.6%	12.6%	1.0%	8.5%	14.1%	3.5%	NR	15.1%
Peru, 1999–2005[27]	794	H3 16.4%	T1 12.3%	18.7%	NR	NR	5.5%	2.8%	NR	3.4%
		H1 3.9%		LAM3 8.3%						
				LAM9 7.4%						
Venezuela, 1998–1999 [38]	670	3%	13%	74%	1.0%	NR	0%	NR	NR	9%
Venezuela, 1997–2006 [40]	1298	5%	10.6%	53%	1.9%	9.7%	0.4%	1.2%	0.2%	17.9%
Bolivia, 2010 [30]	99	39.4%	22.2%	26.3%	2%	NR	NR	1%	NR	9.1%
		H3 30.3%	T1 21.2%	LAM3 15.2%						

A particular characteristic that has been ascribed to the LAM family is that they are unique to harbor a significant proportion of strains with a specific deletion named RD^Rio^ characterized by a single long sequence polymorphism (>26.3 kb) with deletion or modification of 10 genes [28]. LAM1 (SIT20) and LAM2 (SIT17) are exclusively of the RD^Rio^ genotype, LAM4, LAM5, LAM6 and LAM9 are composed of both RD^Rio^ and wild type genotypes, and LAM3 composed exclusively of wild type isolates. This RD^Rio^ genotype was found in 26.5% (72/270) of all tuberculosis cases in a recent large retrospective study from Brazil, extrapolating that this genotype could be widely spread in that country. RD^Rio^ *M*. *tuberculosis* genotypes were suspected of being more virulent and able to cause disease more efficiently, however, not all researchers have supported this view, and a large survey found disease caused by this strain was not clinically distinctive or more severe than disease caused by non-RD^Rio^ [29].

In contrast with other Latin-American countries where LAM is the most prevalent lineage, a shift towards increased proportion of Haarlem lineage was observed in a recent survey of 199 isolates carried out in the North-eastern part of Lima, Peru, where H strains were found in a predominant proportion of 29.6%, followed by T at 15.6%, Beijing at 14.1% and LAM at only 12.6% [26]. Similarly, Bolivia also recently reported a predominance of H genotype in up to 40% of isolates studied in 2010. [30] This Haarlem genotype has the particular significance of having been involved in several outbreaks of MDR-tuberculosis [31] [32]. Furthermore, a recent study conducted in Colombia with strains isolated between 2005 and 2008 has also shown an increase in Haarlem strains actually reaching 44.3% in the city of Medellín, and included a large proportion of MDR among strains belonging to SIT45/H1 [33].

Descriptions for main circulating strains in South-American countries have long described the Euro-American lineage to be widely predominant, and this has been used to support a European dissemination from either early settlement or trade associations [34]. It is of interest to note that this model, however, has been unable to explain the profuse archaeological evidence for the presence of tuberculosis in the Americas before European contact and recent comparative genomics from 1,000-year-old mycobacterial found in Peruvian human skeletons described members of the MTBC most closely related to those adapted to seals and sea lions than those from humans [35]. Worldwide, lineage-specific differences in the virulence and transmissibility of clinical isolates have been reported across independent experimental systems with modern lineages, such as Beijing and Euro-American Haarlem strains believed to exhibit more virulent phenotypes compared with ancient lineages, such as East-African-Indian and *M*. *africanum* [36]. Strains belonging to the Beijing genotype have been associated with increased transmission risk, large outbreaks and drug resistant TB in many parts of the world. This genotype was only recently described for the first time in Chile and is infrequently observed in TB patients in South America with the exception of Peru [37]. On one hand, in Argentina, Brazil, Colombia, Chile, Ecuador, Paraguay and Venezuela, the prevalence of Beijing lineage strains reported has been generally lesser than 2% [21] [23] [28] [37] [38] [39] [40] [41]. On the other hand, the prevalence of Beijing genotype in Peru is exceptionally high and has been estimated between 4.9% and 14.1%, although not clearly associated with MDR tuberculosis [24] [25] [26] [27]. Ritacco et al. speculated that the Beijing lineage strains were first introduced into Peru, and eventually into other South American countries, when Peru received a significant number of Chinese immigrants in the mid-19th century. The need for vigilance is further warranted by another recently published study that included a total of 200 Beijing strains from Peru where 24 loci VNTR analysis showed a high clustering rate (80.3%) and a high recent transmission index (RTI_n−1_ = 0.707), strongly suggesting active and on-going transmission of Beijing lineage strains in the surveyed area [42].

In the present study, susceptibility data was only available for a minority of isolated strains, as susceptibility determination was not required routinely for newly acquired tuberculosis due to the general low MDR rate for the country. With regard to the possibility of strains-other than Beijing- potentially associated with drug resistance, it is of interest to note that in a recent large survey of phylogenetic clades collected over a seventeen-year period in the French Department of the Americas, X and LAM lineages were overrepresented in drug-resistant and MDR-TB cases, respectively, and also, four predominant spoligotypes were significantly associated with drug resistance corresponding to SIT20/LAM1, SIT64/LAM6, SIT45/H1 and SIT46/undefined lineage [43].

The effects of migration on the epidemiology of tuberculosis has been evaluated by many studies by determining genotypic lineages on the isolates and also providing general information on the phylogeographical origin of the strains circulating in the study area [44] [45]. In the present study, 11.6% of strains belonged to migrant population, which is a slightly higher proportion in relation to the national reports (7%), but consistent with our patients who were recruited in the central area of Santiago where the highest immigration rate is found [46]. Among this group, the most prevalent *M*. *tuberculosis* lineage found was also LAM, although infrequently isolated strains such as CAS—belonging to a migrant from Nepal—and a Beijing strain belonging to a migrant from Peru were also detected. As there is considerable evidence that genetic susceptibility can influence on tuberculosis acquisition risk, such as the higher susceptibility conferred by individual polymorphisms in genes for natural resistance-associated macrophage protein (NRAMP1), vitamin D receptor, or mannose-binding protein [47] [48], it has been suggested that different ethnic groups may have different susceptibility for particular *M*. *tuberculosis* strains [49]. This will be an important issue to further explore in Chile and among other populations from Latin America where different ethnic backgrounds coexist and where furthermore, most of ethnic groups are concentrated in areas of poverty and crowding, adding strong confounding factors that can determine a higher prevalence of tuberculosis disease among the most vulnerable.

Finally, it is important to mention that both genotyping methods used in present study are based on analysis of a limited number of loci, and it has been described that VNTR loci as well as particular spoligotyping patterns can exhibit variable discriminatory power in different mycobacterial lineages. Rapid markers evolution with a tendency to converge can lead to significant amounts of homoplasy, and hence the limitations of these tools in defining deep phylogenetic groups in *M*. *tuberculosis* complex or other bacteria [50]. This can explain discordant findings for T and H lineages classification between both SITVIT2 and MIRU-VNTRplus databases (S2 Table). Future investigations by use of more discriminatory tools such as whole genome sequencing (WGS) might be helpful to identify true phylogenetic relationships and also obtain a more precise evaluation of TB transmission.

Among orphan isolates, we noted similar spoligotyping patterns (with absence of spacer 6) among orphans Or08, Or11, and Or12 within our study (S1 Table). The ancestral genotypes found among orphan patterns in our study are most probably suggestive of ancestral lineages undergoing extinction after an initial distant contact with patient(s) infected with Africanum lineage or the African continent. This finding may suggest a probable ancient link with Africanum lineage and the African continent. Interestingly thanks to the “SpolSimilaritySearch tool” under development in SITVIT2 database, we detected that a similar spoligotype was found once in Italy: ■■■■■□■□□□□□■■■■■■■■□□□□■■■■■■■■■■■■□□□■■■■; octal code 764077607777071; AFRI_2. However, one cannot exclude the possibility of a recent introduction of such strains in Chile.

A supplemental figure (S2 Fig.) has been added to support the finding of high presence of SIT37/T3 in Chile. This figure shows geographical maps based on SITVIT2 interrogation prior to entry of present dataset to summarize percentages of SIT37 (A) and strains belonging to T3 sub-lineage (B), by country. Note that this distribution should not be confounded with data in Table 3, which shows proportions of a given spoligotype in the global database. For example, we have 7 strains for SIT37/T3 representing 6.8% of our study sample. However when checked in the database, these 7 strains represent 1.34% of all SIT37/T3 strains present in SITVIT2. Finally, if we look for all identical strains in the database, we see that 19.19% were reported from AFRI-E, 11.9% from EURO-N, 11.52% from EURO-W, etc. Finally, we have also added a new map (S3 Fig.) to show the global distribution of lineages in USA, Canada, and various Latin American and Caribbean countries as compared to Chile.

In conclusion, we have shown the predominance of LAM and T genotypes corresponding to almost two thirds of circulating stains for the central area of Santiago, Chile, in the study period. We have also underlined the significant presence of SIT37/T3 among Chilean isolates. This high proportion of SIT37/T3 in our study in Chile as well as in other countries of Latin America (Guyana, Cuba, or Dominican Republic) in conjunction with East Africa, and to a lesser extent in other regions and countries—including not negligible proportions in Nepal and China—suggests long-lasting spread of this genotype. Obviously, more data and analysis are needed to clarify probable ancestral human migrations responsible for its actual distribution pattern. Further studies on a larger number of isolates and from patients with larger geographic and ethnic diversity might help to accurately estimate the genetic landscape of prevailing *M*. *tuberculosis* epidemic in Chile.

## Supporting Information

S1 Fig Unweighted Pair Group Method with Arithmetic Mean (UPGMA) tree based on the combination of spoligotypes and 12-loci MIRUs (using MLVA Compare software). Labels identifying all strains are as follows: strain number, lineage, SIT, 12-MIT.
(TIF)

S2 Fig Geographical maps (obtained by interrogating SITVIT2 database prior to entry of this study) representing percentage distributions of strains belonging to SIT37 (A), and strains belonging to T3 sub-lineage (B). Maps were reproduced from work created and shared by Google (https://google-developers.appspot.com/site-policies) and used according to terms described in the Creative Commons 3.0 Attribution License (http://creativecommons.org/licenses/by/3.0/).
(TIF)

S3 Fig Geographic distribution of lineages in Latin American and neighboring countries. Note that the map file was downloaded under Creative Commons License using the link: http://en.wikipedia.org/wiki/Latin_America and was manually modified for representative purposes only.
(TIF)

S1 Table Description of spoligotypes and MIRU12 patterns corresponding to orphans strains (n = 16) in the SITVIT2 database. (DOCX)

S2 Table Description of spoligotypes, MIRU12 patterns and lineage allocation by SITVIT2 and MIRU-VNTRplus for all the isolates of present study (n = 103). (PDF)
