# Supplementary figures and images for: A First Insight on the Population Structure of Mycobacterium tuberculosis Complex as Studied by Spoligotyping and MIRU-VNTRs in Santiago, Chile
Source: PLoS One. 2015 Feb 11;10(2):e0118007. doi: 10.1371/journal.pone.0118007 (PMC4324903; doi:10.1371/journal.pone.0118007)

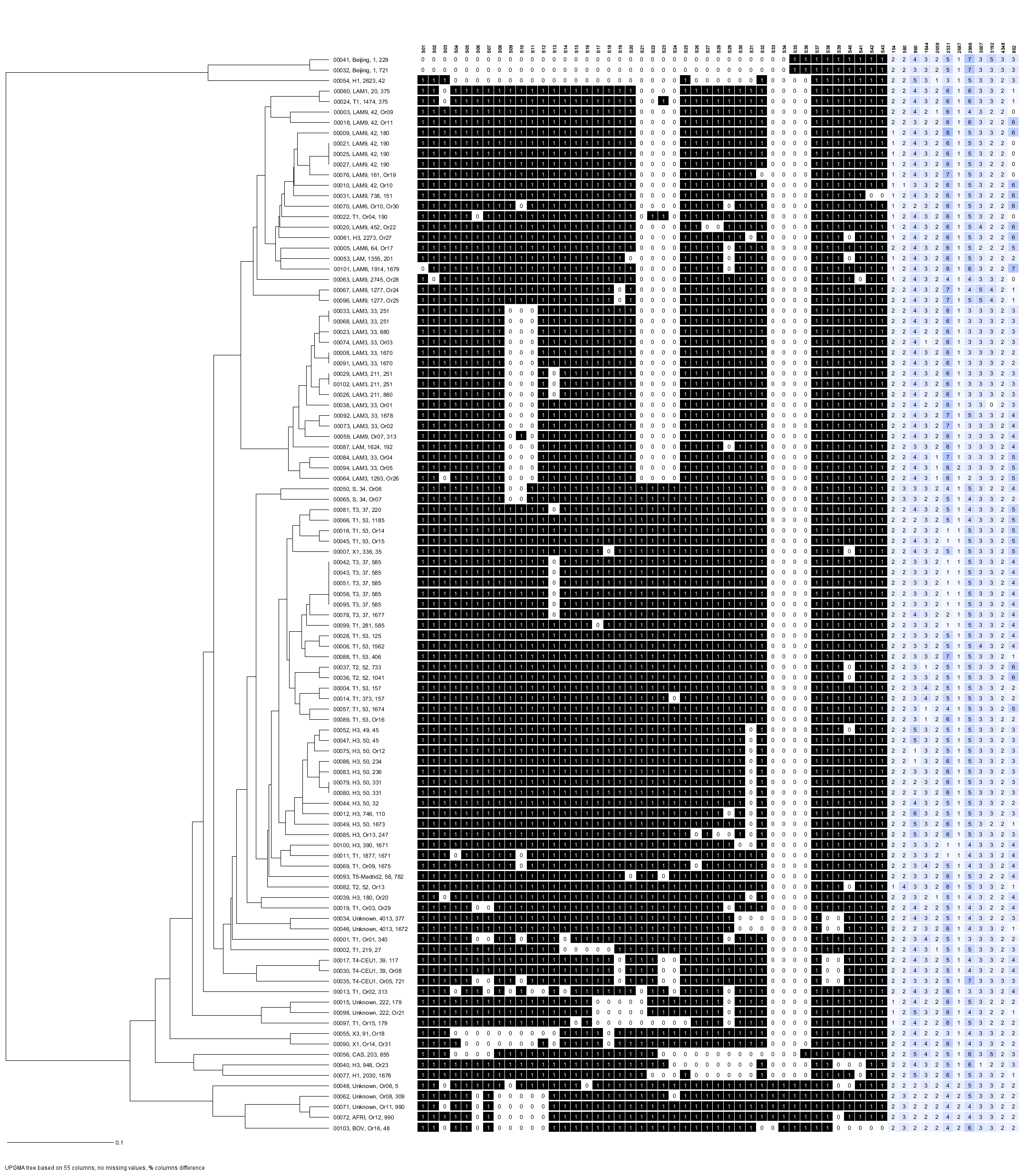

Supplement: S1 Fig — Labels identifying all strains are as follows: strain number, lineage, SIT, 12-MIT. (TIF) [file pone.0118007.s001.tif]

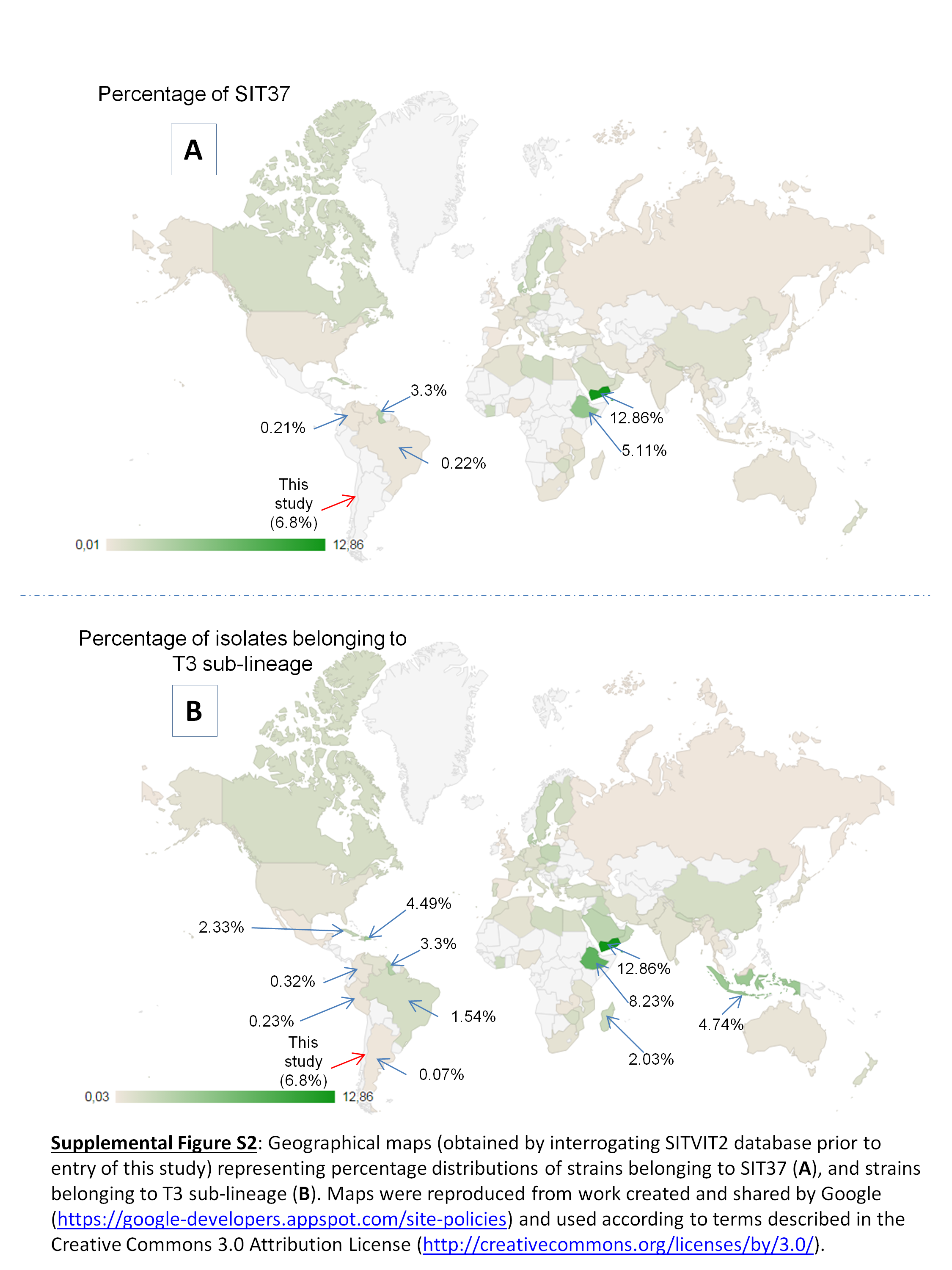

Supplement: S2 Fig — Maps were reproduced from work created and shared by Google (https://google-developers.appspot.com/site-policies) and used according to terms described in the Creative Commons 3.0 Attribution License (http://creativecommons.org/licenses/by/3.0/). (TIF) [file pone.0118007.s002.tif]

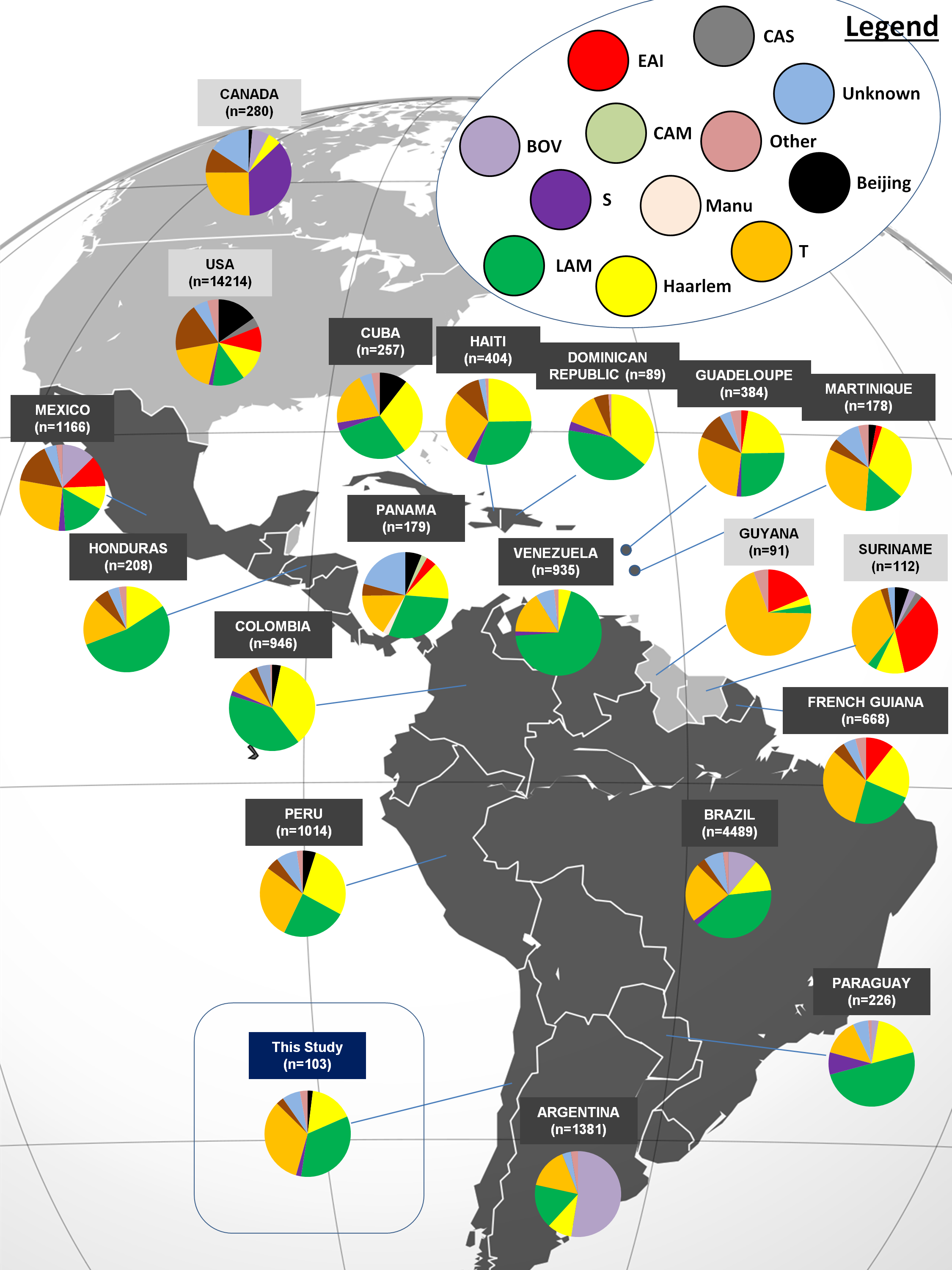

Supplement: S3 Fig — Note that the map file was downloaded under Creative Commons License using the link: http://en.wikipedia.org/wiki/Latin_America and was manually modified for representative purposes only. (TIF) [file pone.0118007.s003.tif]
