# Supplementary material for: A First Insight on the Population Structure of Mycobacterium tuberculosis Complex as Studied by Spoligotyping and MIRU-VNTRs in Santiago, Chile
Source: PLoS One. 2015 Feb 11;10(2):e0118007. doi: 10.1371/journal.pone.0118007 (PMC4324903; doi:10.1371/journal.pone.0118007)
Supplement: S1 Table — (DOCX) [file pone.0118007.s004.docx]

| **IsoNumber** | **Spoligotype Description** | **Octal code** | **Sublineage** | **Lineage** | **SIT** | **MIRU12** | **12-MIT** |
| --- | --- | --- | --- | --- | --- | --- | --- |
| CHL022008000001 | ■■■■■□□■■□■■■□■■■■■■■■■■■■■■□■■■□□□□■■■■■■■ | 763357777560771 | T1 | T | Or01 | 223425133322 | 340 |
| CHL022010300013 | ■■■□■■□■□■□□■□■■■■■■□■■□■■■■□■■■□□□□■■■■■■■ | 732457667560771 | T1 | T | Or02 | 224326133324 | 313 |
| CHL022011000019 | ■■■■■□□■■■■■■■■■■■■■■■■■■■■■□■■■□□□□■■■■■■■ | 763777777560771 | T1 | T | Or03 | 224225143224 | Or29 |
| CHL022011000022 | ■■■■■□■■■■■■■■■■■■■■□■■□■■■■■■■■□□□□■■■■■■■ | 767777667760771 | T1 | T | Or04 | 124326153220 | 190 |
| CHL022011000035 | ■■■■■□□■■□■■■■■■■■□■■■□□■■■■■■■■□□□□■□□■■■■ | 763377347760471 | T4-CEU1 | T | Or05 | 223325173333 | 721 |
| CHL022013300048 | ■■□■■■■■□■■■■■■□■■■■■■■■■■■■■■■■■■■■■■□□■■■ | 676773777777631 | Unknown | Unknown | Or06 | 222324253322 | 5 |
| CHL022012000059 | ■■■■■■■■□■□■■■■■■■■■□□□□■■■■■■■■□□□□■■■■■■■ | 776577607760771 | LAM9 | LAM | Or07 | 224326133324 | 313 |
| CHL022012100062 | ■■■■■□■□□□□□■■■■■■■■■■■□■■■■■■■■■■■■■■■■■■■ | 764077767777771 | Unknown | Unknown | Or08 | 232224253322 | 309 |
| CHL022012100069 | ■■■■■■■■■□■■■■■■■■■■■■■■■□■■■■■■□□□□■■■■■■■ | 777377775760771 | T1 | T | Or09 | 223425143324 | 1675 |
| CHL022012000070 | ■■■■■■■■■□■■■■■■■■■■□□□□■■■■□■■■□□□□■■■■■■■ | 777377607560771 | LAM6 | LAM | Or10 | 122326153226 | Or30 |
| CHL022012100071 | ■■□■■□■□□□□□■■■■■■■■■■■■■■■■■■■■■■■■■■■■■■■ | 664077777777771 | Unknown | Unknown | Or11 | 232224243322 | 990 |
| CHL022012100072 | ■■■■■□■□□□□□■■■■■■■■■■■■■■■■■■■■■■■■■■□■■■■ | 764077777777671 | AFRI | AFRI | Or12 | 232224243322 | 990 |
| CHL022013100085 | ■■■■■■■■■■■■■■■■■■■■■■■■■□■□□■□■□□□□■■■■■■■ | 777777775120771 | H3 | H | Or13 | 225326153323 | 247 |
| CHL022013000090 | ■■■■□□□□□□□■□■■■■□■■■■■■■■■■■■■■□□□□■■■■■■■ | 740136777760771 | X1 | X | Or14 | 224426143322 | Or31 |
| CHL022013000097 | ■■■■■■■■■■■■■■□■□□□□□□□□□■■■□■■■□□□□■■■■■■■ | 777764003560771 | T1 | T | Or15 | 124226153222 | 179 |
| CHL022013000103 | ■■□■■□■□□□□□■■■■■■■■■■■■■■■■■■■□□■■■■■□□□□□ | 664077777747600 | BOV | BOV | Or16 | 232224263322 | 48 |

**Supplemental Table S1.** Description of spoligotypes and MIRU12 patterns (n=16) corresponding to orphans strains in the SITVIT2 database.
