# Supplementary material for: A First Insight on the Population Structure of Mycobacterium tuberculosis Complex as Studied by Spoligotyping and MIRU-VNTRs in Santiago, Chile
Source: PLoS One. 2015 Feb 11;10(2):e0118007. doi: 10.1371/journal.pone.0118007 (PMC4324903; doi:10.1371/journal.pone.0118007)
Supplement: S2 Table — (PDF) [file pone.0118007.s005.pdf]

IsoNumber	Year	Spoligotype Description	Octal code	Sublineage	Lineage	SIT	MIRU12	12-MIT	Lineage (MIRU-VNTR plus)	Sex / Age
CHL022011000041	2011	□□□□□□□□□□□□□□□□□□□□□□□□□□□□□□□□□□■■■■■■■■■	000000000003771	Beijing	Beijing	1	224325173533	229	Beijing	M/56
CHL022011000032	2011	□□□□□□□□□□□□□□□□□□□□□□□□□□□□□□□□□□■■■■■■■■■	000000000003771	Beijing	Beijing	1	223325173333	721	Beijing	F/91
CHL022012000060	2012	■■□■■■■■■■■■■■■■■■■■□□□□■■■■■■■■□□□□■■■■■■■	677777607760771	LAM1	LAM	20	224326163321	375	LAM	M
CHL022011300033	2011	■■■■■■■■□□□■■■■■■■■■□□□□■■■■■■■■□□□□■■■■■■■	776177607760771	LAM3	LAM	33	224326133323	251	LAM	M/31
CHL022012100068	2012	■■■■■■■■□□□■■■■■■■■■□□□□■■■■■■■■□□□□■■■■■■■	776177607760771	LAM3	LAM	33	224326133323	251	LAM	M/24
CHL022011000023	2011	■■■■■■■■□□□■■■■■■■■■□□□□■■■■■■■■□□□□■■■■■■■	776177607760771	LAM3	LAM	33	224226133323	880	LAM	F/29
CHL022010000008	2010	■■■■■■■■□□□■■■■■■■■■□□□□■■■■■■■■□□□□■■■■■■■	776177607760771	LAM3	LAM	33	224326133322	1670	LAM	F/63
CHL022013000091	2013	■■■■■■■■□□□■■■■■■■■■□□□□■■■■■■■■□□□□■■■■■■■	776177607760771	LAM3	LAM	33	224326133322	1670	LAM	M/27
CHL022013000092	2013	■■■■■■■■□□□■■■■■■■■■□□□□■■■■■■■■□□□□■■■■■■■	776177607760771	LAM3	LAM	33	224327153324	1678	LAM	F/29
CHL022011000038	2011	■■■■■■■■□□□■■■■■■■■■□□□□■■■■■■■■□□□□■■■■■■■	776177607760771	LAM3	LAM	33	224226133023	Or01	LAM	F/36
CHL022013000073	2013	■■■■■■■■□□□■■■■■■■■■□□□□■■■■■■■■□□□□■■■■■■■	776177607760771	LAM3	LAM	33	224327133324	Or02	LAM	M/56
CHL022013000074	2013	■■■■■■■■□□□■■■■■■■■■□□□□■■■■■■■■□□□□■■■■■■■	776177607760771	LAM3	LAM	33	224126133323	Or03	LAM	M/31
CHL022013000084	2013	■■■■■■■■□□□■■■■■■■■■□□□□■■■■■■■■□□□□■■■■■■■	776177607760771	LAM3	LAM	33	224317133325	Or04	LAM	F/53
CHL022013000094	2013	■■■■■■■■□□□■■■■■■■■■□□□□■■■■■■■■□□□□■■■■■■■	776177607760771	LAM3	LAM	33	224316233325	Or05	LAM	F/27
CHL022012000050	2012	■■■■■■■■□□■■■■■■■■■■■■■■■■■■■■■■□□□□■■■■■■■	776377777760771	S	S	34	233324153224	Or06	S	M/59
CHL022012100065	2012	■■■■■■■■□□■■■■■■■■■■■■■■■■■■■■■■□□□□■■■■■■■	776377777760771	S	S	34	233225143322	Or07	S	M/45
CHL022013000081	2013	■■■■■■■■■■■■□■■■■■■■■■■■■■■■■■■■■■■□□□□■■■■■■■	777737777760771	T3	T	37	224325143325	220	X	F/60
CHL022013100042	2013	■■■■■■■■■■■■□■■■■■■■■■■■■■■■■■■■■■■□□□□■■■■■■■	777737777760771	T3	T	37	223321153324	585	H	M/52
CHL022013100043	2013	■■■■■■■■■■■■□■■■■■■■■■■■■■■■■■■■■■■□□□□■■■■■■■	777737777760771	T3	T	37	223321153324	585	H	F/51
CHL022012000051	2012	■■■■■■■■■■■■□■■■■■■■■■■■■■■■■■■■■■■□□□□■■■■■■■	777737777760771	T3	T	37	223321153324	585	H	M/3
CHL022012000058	2012	■■■■■■■■■■■■□■■■■■■■■■■■■■■■■■■■■■■□□□□■■■■■■■	777737777760771	T3	T	37	223321153324	585	H	F
CHL022013000095	2013	■■■■■■■■■■■■□■■■■■■■■■■■■■■■■■■■■■■□□□□■■■■■■■	777737777760771	T3	T	37	223321153324	585	H	M/50
CHL022013000078	2013	■■■■■■■■■■■■□■■■■■■■■■■■■■■■■■■■■■■□□□□■■■■■■■	777737777760771	T3	T	37	224322153324	1677	H	M
CHL022011000017	2011	■■■■■■■■■■■■■■■■■■□■■■□□■■■■■■■■□□□□■□□■■■■	777777347760471	T4-CEU1	T	39	224325143324	117	X	M/28
CHL022011000030	2011	■■■■■■■■■■■■■■■■■■□■■■□□■■■■■■■■□□□□■□□■■■■	777777347760471	T4-CEU1	T	39	224325143224	Or08	LAM	M/29
CHL022010000009	2010	■■■■■■■■■■■■■■■■■■■■□□□□■■■■■■■■□□□□■■■■■■■	777777607760771	LAM9	LAM	42	124328153326	180	LAM	F/93
CHL022011000021	2011	■■■■■■■■■■■■■■■■■■■■□□□□■■■■■■■■□□□□■■■■■■■	777777607760771	LAM9	LAM	42	124326153220	190	LAM	F/42
CHL022011000025	2011	■■■■■■■■■■■■■■■■■■■■□□□□■■■■■■■■□□□□■■■■■■■	777777607760771	LAM9	LAM	42	124326153220	190	LAM	M/88
CHL022011000027	2011	■■■■■■■■■■■■■■■■■■■■□□□□■■■■■■■■□□□□■■■■■■■	777777607760771	LAM9	LAM	42	124326153220	190	LAM	M/44
CHL022009100003	2009	■■■■■■■■■■■■■■■■■■■■□□□□■■■■■■■■□□□□■■■■■■■	777777607760771	LAM9	LAM	42	224216143220	Or09	LAM	F/74
CHL022009000010	2009	■■■■■■■■■■■■■■■■■■■■□□□□■■■■■■■■□□□□■■■■■■■	777777607760771	LAM9	LAM	42	113326153226	Or10	LAM	F/50

CHL022011000018	2011	■■■■■■■■■■■■■■■■■■■■□□□□■■■■■■■■□□□□■■■■■■■	777777607760771	LAM9	LAM	42	223226163226	Or11	LAM	M/66
CHL022012000052	2012	■■■■■■■■■■■■■■■■■■■■■■■■■■■■■■□■□□□□■■■□■■■	777777777720731	H3	H	49	225325153323	45	H	M/24
CHL022013100044	2013	■■■■■■■■■■■■■■■■■■■■■■■■■■■■■■□■□□□□■■■■■■■	777777777720771	H3	H	50	224325153322	32	H	M/ 35
CHL022013100047	2013	■■■■■■■■■■■■■■■■■■■■■■■■■■■■■■□■□□□□■■■■■■■	777777777720771	H3	H	50	225325153323	45	H	M/44
CHL022013100086	2013	■■■■■■■■■■■■■■■■■■■■■■■■■■■■■■□■□□□□■■■■■■■	777777777720771	H3	H	50	221326153323	234	H	M/68
CHL022013000083	2013	■■■■■■■■■■■■■■■■■■■■■■■■■■■■■■□■□□□□■■■■■■■	777777777720771	H3	H	50	223326153323	236	H	M/39
CHL022013000079	2013	■■■■■■■■■■■■■■■■■■■■■■■■■■■■■■□■□□□□■■■■■■■	777777777720771	H3	H	50	222326153323	331	H	M/20
CHL022013000080	2013	■■■■■■■■■■■■■■■■■■■■■■■■■■■■■■□■□□□□■■■■■■■	777777777720771	H3	H	50	222326153323	331	H	M/29
CHL022012000049	2012	■■■■■■■■■■■■■■■■■■■■■■■■■■■■■■□■□□□□■■■■■■■	777777777720771	H3	H	50	225326153221	1673	H	M/52
CHL022013000075	2013	■■■■■■■■■■■■■■■■■■■■■■■■■■■■■■□■□□□□■■■■■■■	777777777720771	H3	H	50	221325153323	Or12	H	M/25
CHL022011000037	2011	■■■■■■■■■■■■■■■■■■■■■■■■■■■■■■■■□□□□■■■□■■■	777777777760731	T2	T	52	223125153326	733	S	F/71
CHL022011000036	2011	■■■■■■■■■■■■■■■■■■■■■■■■■■■■■■■■□□□□■■■□■■■	777777777760731	T2	T	52	223325153326	1041	Ghana	F/61
CHL022013000082	2013	■■■■■■■■■■■■■■■■■■■■■■■■■■■■■■■■□□□□■■■□■■■	777777777760731	T2	T	52	143326153321	Or13	unknown	F/67
CHL022011000028	2011	■■■■■■■■■■■■■■■■■■■■■■■■■■■■■■■■□□□□■■■■■■■	777777777760771	T1	T	53	223325153324	125	S	F/30
CHL022009000004	2009	■■■■■■■■■■■■■■■■■■■■■■■■■■■■■■■■□□□□■■■■■■■	777777777760771	T1	T	53	223425153322	157	Ugandal	F/74
CHL022013100088	2013	■■■■■■■■■■■■■■■■■■■■■■■■■■■■■■■■□□□□■■■■■■■	777777777760771	T1	T	53	223327153321	406	H	M/53
CHL022012300066	2012	■■■■■■■■■■■■■■■■■■■■■■■■■■■■■■■■□□□□■■■■■■■	777777777760771	T1	T	53	222325143325	1185	Ghana	M/63
CHL022010000006	2010	■■■■■■■■■■■■■■■■■■■■■■■■■■■■■■■■□□□□■■■■■■■	777777777760771	T1	T	53	223325154324	1562	S	M/66
CHL022012000057	2012	■■■■■■■■■■■■■■■■■■■■■■■■■■■■■■■■□□□□■■■■■■■	777777777760771	T1	T	53	223124153325	1674	H	M/28
CHL022010000016	2010	■■■■■■■■■■■■■■■■■■■■■■■■■■■■■■■■□□□□■■■■■■■	777777777760771	T1	T	53	222321153325	Or14	H	F/76
CHL022013100045	2013	■■■■■■■■■■■■■■■■■■■■■■■■■■■■■■■■□□□□■■■■■■■	777777777760771	T1	T	53	224321153325	Or15	X	M/50
CHL022013100089	2013	■■■■■■■■■■■■■■■■■■■■■■■■■■■■■■■■□□□□■■■■■■■	777777777760771	T1	T	53	223126153322	Or16	LAM	M/5
CHL022013000093	2013	■■■■■■■■■■■■■■■■■■■□■■□■■■■■■■■■■□□□□■■■■■■■	777777557760771	T5-Madrid2	T	58	223326153224	782	H	F/22
CHL022009000005	2009	■■■■■■■■■■■■■■■■■■■■□□□□■■■■□■■■□□□□■■■■■■■	777777607560771	LAM6	LAM	64	224326152225	Or17	LAM	F/43
CHL022012000055	2012	■■■□□□□□□□□□□■■■■□■■■■■■■■■■■■■■□□□□■■■■■■■	700036777760771	X3	X	91	224223143322	Or18	unknown	F/38
CHL022013000076	2013	■■■■■■■■■■■■■■■■■■■■□□□□■■■■■■■□□□□□■■■■■■■	777777607740771	LAM9	LAM	161	124327153220	Or19	LAM	M/37
CHL022011000039	2011	■■□■■■■■■■■■■■■■■■■■■■■■■■■■■■□■□□□□■■■■■■■	677777777720771	H3	H	180	223215143224	Or20	Ugandal	F/29
CHL022012000056	2012	■■■□□□□■■■■■■■■■■■■■■■□□□□□□□□□□□□□■■■■■■■■	703777740001771	CAS	CAS	203	225425163523	855	Delhi/CAS	F/38
CHL022011000029	2011	■■■■■■■■□□□■□■■■■■■■□□□□■■■■■■■■□□□□■■■■■■■	776137607760771	LAM3	LAM	211	224326133323	251	LAM	F/83
CHL022013000102	2013	■■■■■■■■□□□■□■■■■■■■□□□□■■■■■■■■□□□□■■■■■■■	776137607760771	LAM3	LAM	211	224326133323	251	LAM	M/49
CHL022011000026	2011	■■■■■■■■□□□■□■■■■■■■□□□□■■■■■■■■□□□□■■■■■■■	776137607760771	LAM3	LAM	211	224226133323	880	LAM	M/74
CHL022009000002	2009	■■■■■■■■■■■■■□□□□□■■■■■■■■■■■■■■□□□□■■■■■■■	777740777760771	T1	T	219	224315153323	27	Cameroon	F/74
CHL022010000015	2010	■■■■■■■■■■■■■■■■□□□□□■■■■■■■□■■■□□□□■■■■■■■	777774077560771	Unknown	Unknown	222	124226153222	179	LAM	M/36
CHL022013000098	2013	■■■■■■■■■■■■■■■■□□□□□■■■■■■■□■■■□□□□■■■■■■■	777774077560771	Unknown	Unknown	222	125326143221	Or21	unknown	M/56

CHL022013000099	2013	■■■■■■■■■■■■■■■■□■■■■■■■■■■■■■■■□□□□■■■■■■■	777775777760771	T1	T	281	223321153324	585	H	M/46
CHL022010000007	2010	■■■■■■■■■■■■■■■■■□■■■■■■■■■■■■■■□□□□■■■□■■■	777776777760731	X1	X	336	224325153325	35	X	F/38
CHL022010000014	2010	■■■■■■■■■■■■■■■■■■■■■■■□■■■■■■■■□□□□■■■■■■■	777777767760771	T1	T	373	223425153322	157	Ugandal	M/61
CHL022013100100	2013	■■■■■■■■■■■■■■■■■■■■■■■■■■■■■□□■□□□□■■■■■■■	777777777620771	H3	H	390	223321143324	1671	H	F/25
CHL022011000020	2011	■■■■■■■■■■■■■■■■■■■■□□□□■■□□■■■■□□□□■■■■■■■	777777606360771	LAM9	LAM	452	124326154226	Or22	LAM	M/31
CHL022011000031	2011	■■■■■■■■■■■■■■■■■■■■□□□□■■■■■■■■□□□□■■■■■□□	777777607760760	LAM9	LAM	738	124326153226	151	LAM	F/73
CHL022010000012	2010	■■■■■■■■■■■■■■■■■■■■■■■■■■■■□■□■□□□□■■■■■■■	777777777520771	H3	H	746	226325153323	110	H	M/66
CHL022011000040	2011	■■■■■■■■■■■■■■■■■■■■■■■□□□□□□□□■□□□□■■□□□■■	777777760020611	H3	H	948	224325161223	Or23	unknown	F/51
CHL022012100067	2012	■■■■■■■■■■■■■■■■■■□■□□□□■■■■■■■■□□□□■■■■■■■	777777207760771	LAM9	LAM	1277	224327145421	Or24	unknown	M/32
CHL022013000096	2013	■■■■■■■■■■■■■■■■■■□■□□□□■■■■■■■■□□□□■■■■■■■	777777207760771	LAM9	LAM	1277	224327155421	Or25	LAM	M/39
CHL022012100064	2012	■■□■■■■■□□□■■■■■■■■■□□□□■■■■■■■■□□□□■■■■■■■	676177607760771	LAM3	LAM	1293	224316123325	Or26	LAM	F/25
CHL022012000053	2012	■■■■■■■■■■■■■■■■■■■□□□□□■■■■□■■■□□□□■■■□■■■	777777407560731	LAM	LAM	1355	124326153222	201	LAM	M/16
CHL022011000024	2011	■■□■■■■■■■■■■■■■■■■■□□■□■■■■■■■■□□□□■■■■■■■	677777627760771	T1	T	1474	224326163321	375	LAM	M/71
CHL022013100087	2013	■■■■■■■■□□□■■■■■■■■■□□□□■■■■□■■■□□□□■■■■■■■	776177607560771	LAM	LAM	1624	223326133324	192	LAM	F
CHL022009000011	2009	■■■□■■■■■□■■■■■■■■■■■■■■■■■■■■■■□□□□■■■■■■■	737377777760771	T1	T	1877	223321143324	1671	Ghana	F/37
CHL022013000101	2013	□■■■■■■■■■■■■■■■■■■■□□□□■■■■□■■■□□□□■■■■■■■	377777607560771	LAM6	LAM	1914	124326163227	1679	LAM	M/48
CHL022013000077	2013	■■■■■■■■■■■■■■■■■■■■■□□□■□□□□□□■□□□□■■■■□■■	777777704020751	H1	H	2030	225326153321	1676	unknown	M/34
CHL022012000061	2012	■■■■■■■■■■■■■■■■■■■■□□□□■■■■■■□■□□□□■■■□■■■	777777607720731	H3	H	2273	124226153226	Or27	LAM	M/24
CHL022012000054	2012	■■■□□□□□□□□□□□□□□□□□□□□□■□□□□□□■□□□□■■■■■■■	700000004020771	H1	H	2623	225313153323	42	H	M/46
CHL022012000063	2012	■□■■■■■■■■■■■■■■■■■■□□□□■■■■■■■■□□□□■■■■□■■	577777607760751	LAM9	LAM	2745	124324143320	Or28	LAM	M/31
CHL022011200034	2011	■■■■■■■■■■■■■■■■■■■■■■■■■■■■■□□□□□□□■□□■■■■	777777777600471	Unknown	Unknown	4013*	224325143223	377	unknown	M/40
CHL022012000046	2012	■■■■■■■■■■■■■■■■■■■■■■■■■■■■■□□□□□□□■□□■■■■	777777777600471	Unknown	Unknown	4013*	222326143321	1672	unknown	M/67
CHL022008000001	2008	■■■■■□□■■□■■■□■■■■■■■■■■■■■■□■■■□□□□■■■■■■■	763357777560771	T1	T	Or01	223425133322	340	unknown	F/74
CHL022010300013	2010	■■■□■■□■□■□□■□■■■■■■□■■□■■■■□■■■□□□□■■■■■■■	732457667560771	T1	T	Or02	224326133324	313	LAM	F/37
CHL022011000019	2011	■■■■■□□■■■■■■■■■■■■■■■■■■■■■□■■■□□□□■■■■■■■	763777777560771	T1	T	Or03	224225143224	Or29	Ugandal	M/63
CHL022011000022	2011	■■■■■□■■■■■■■■■■■■■■□■■□■■■■■■■■□□□□■■■■■■■	767777667760771	T1	T	Or04	124326153220	190	LAM	F/33
CHL022011000035	2011	■■■■■□□■■□■■■■■■■■□■■■□□■■■■■■■■□□□□■□□■■■■	763377347760471	T4-CEU1	T	Or05	223325173333	721	unknown	F/36
CHL022013300048	2013	■■□■■■■■□■■■■■■□■■■■■■■■■■■■■■■■■■■■■□□■■■	676773777777631	Unknown	Unknown	Or06	222324253322	5	Bovis	F/84
CHL022012000059	2012	■■■■■■■■□■□■■■■■■■■■□□□□■■■■■■■■□□□□■■■■■■■	776577607760771	LAM9	LAM	Or07	224326133324	313	LAM	M/19
CHL022012100062	2012	■■■■■□■□□□□□■■■■■■■■■■■□■■■■■■■■■■■■■■■■■■■	764077767777771	Unknown	Unknown	Or08	232224253322	309	unknown	F/23
CHL022012100069	2012	■■■■■■■■■□■■■■■■■■■■■■■■■□■■■■■■□□□□■■■■■■■	777377775760771	T1	T	Or09	223425143324	1675	Ghana	M/68
CHL022012000070	2012	■■■■■■■■■□■■■■■■■■■■□□□□■■■■□■■■□□□□■■■■■■■	777377607560771	LAM6	LAM	Or10	122326153226	Or30	LAM	M/45
CHL022012100071	2012	■■□■■□■□□□□□■■■■■■■■■■■■■■■■■■■■■■■■■■■■■■■	664077777777771	Unknown	Unknown	Or11	232224243322	990	unknown	M/63
CHL022012100072	2012	■■■■■□■□□□□□■■■■■■■■■■■■■■■■■■■■■■■■■■□■■■■	764077777777671	AFRI	AFRI	Or12	232224243322	990	unknown	M/93

CHL022013100085	2013	■■■■■■■■■■■■■■■■■■■■■■■■■□■□□■□■□□□□■■■■■■■	777777775120771	H3	H	Or13	225326153323	247	H	M/57
CHL022013000090	2013	■■■■□□□□□□□■□■■■■□■■■■■■■■■■■■■■□□□□■■■■■■■	740136777760771	X1	X	Or14	224426143322	Or31	unknown	M/79
CHL022013000097	2013	■■■■■■■■■■■■■■□■□□□□□□□□□■■■□■■■□□□□■■■■■■■	777764003560771	T1	T	Or15	124226153222	179	LAM	M/22
CHL022013000103	2013	■■□■■□■□□□□□■■■■■■■■■■■■■■■■■■■□□■■■■■□□□□□	664077777747600	BOV	BOV	Or16	232224263322	48	Bovis	M/62

(*) Newly created SIT are highlighted by an asterisk (*)
